# Thermally Driven Self-Rotation of a Hollow Torus Motor

**DOI:** 10.3390/mi13030434

**Published:** 2022-03-12

**Authors:** Changshen Du, Biao Zhang, Quanbao Cheng, Peibao Xu, Kai Li

**Affiliations:** Department of Civil Engineering, Anhui Jianzhu University, Hefei 230601, China; changshendu@yeah.net (C.D.); zhangb029@163.com (B.Z.); cheng_quanbao@outlook.com (Q.C.); peibaoxu@ahjzu.edu.cn (P.X.)

**Keywords:** self-sustained oscillation, thermally responsive, hollow torus, eversion/inversion, energy efficiency

## Abstract

Self-oscillating systems based on thermally responsive polymer materials can realize heat-mechanical transduction in a steady ambient temperature field and have huge application potential in the field of micro-active machines, micro-robotics and energy harvesters. Recently, experiments have found that a torus on a hot surface can rotate autonomously and continuously, and its rotating velocity is determined by the competition between the thermally induced driving moment and the sliding friction moment. In this article, we theoretically study the self-sustained rotation of a hollow torus on a hot surface and explore the effect of the radius ratio on its rotational angular velocity and energy efficiency. By establishing a theoretical model of heat-driven self-sustained rotation, its analytical driving moment is derived, and the equilibrium equation for its steady rotation is obtained. Numerical calculation shows that with the increase in the radius ratio, the angular velocity of its rotation monotonously increases, while the energy efficiency of the self-rotating hollow torus motor first increases and then decreases. In addition, the effects of several system parameters on the angular velocity of it are also extensively investigated. The results in this paper have a guiding role in the application of hollow torus motor in the fields of micro-active machines, thermally driven motors and waste heat harvesters.

## 1. Introduction

Self-excited oscillation is a kind of periodic motion fueled by a constant external stimulation [1,2,3,4] and has potential applications in the areas of motors [5,6,7], micro-active machines [8,9,10,11,12], energy harvester [13,14,15] and micro-robotics [16,17,18,19]. Similar to biological active feeding, self-oscillation can directly harvest energy from a constant environment to maintain its periodic motion [20,21]. This feature makes the self-oscillating system has no requirement of complex controllers or heavy batteries, and is simple and portable [22,23]. In addition, the self-sustained oscillation has robustness, and can ensure the stability and normal operation of various systems based on self-sustained oscillation [24,25]. 

In recent years, based on various stimuli-responsive materials including thermally responsive polymer materials [26,27], liquid crystal elastomers (LCEs) [28,29,30,31], dielectric elastomer [32,33,34,35], hydrogels [36,37], and ion gels [38,39], a wealth of self-excited motion modes have been proposed, such as rolling [40,41], vibration [24,31,40,41], torsion [41,42], stretching and shrinking [10,40,43], swinging [44], buckling [45,46], jumping [47,48,49], rotation [25,28], eversion or inversion [27,50], expansion and contraction [51,52], swimming [53] and even group behavior of several coupled self-excited oscillators [54]. The self-oscillating system is usually accompanied with damping dissipation, and its motion is a non-equilibrium dissipation process. In order to realize and maintain the self-oscillation, various mechanisms for compensating the damping energy dissipation by inputting net energy have been proposed, such as coupling of chemical reaction and large deformation [55], self-shading effect [17,56], multi-process coupling of droplet evaporation and plate bending [57]. 

Recently, a very interesting self-sustained motion with a zero-energy mode was reported, where a nylon torus or PDMS torus sitting on a hot surface can autonomously and continuously rotate [27,50]. During the rotation, the Nylon/PDMS torus continuously converts thermal energy to mechanical work, we can use it as a heat-driven motor or use it to harvest low-grade waste heat. For thermally expanding materials (Nylon) the torus everts, for thermally expanding materials (PDMS) the torus inverts [27]. The rotation angular velocity of the torus is determined by the competition between the thermally driven driving moment and the sliding friction moment of the torus, and the parameters including thermal expansion coefficient, heat flux, contact angle, curvature of the torus, heat transfer coefficient and sliding friction coefficient affect the angular velocity of the torus by influencing the driving moment or the friction moment [25,50]. 

Similar to the torsional properties of the cylindrical rod [58], the center part of the torus contributes little to the driving moment but a lot to the friction moment of the torus, so the hollow torus may have a greater rotation angular velocity than the solid torus. In order to increase the rotation angular velocity of the torus and improve the thermal-mechanical energy conversion efficiency of “toroidal motor” [27], this work will focus on the effect of the radius ratio of the hollow torus on its rotation angular velocity and energy efficiency and try to find an optimal radius ratio. In addition, the effects of the other system parameters on the rotation angular velocity of the hollow torus will also be studied. 

The layout of this paper is as follows: first, a theoretical model of thermally driven self-sustained rotation of a hollow torus on a hot surface is established, and the analytical solution of the driving moment for rotation of the hollow torus is derived in Section 2. Then, the equilibrium equations for the steady self-rotation of the hollow torus are given in Section 3. Meanwhile, the effects of the thermal expansion coefficient, heat flux, contact angle, curvature of the hollow torus, heat transfer coefficient and sliding friction coefficient on the rotation angular velocity of the hollow torus are studied in detail, and the dependence of the energy efficiency on the radius ratio and dimensionless heat flux is also investigated. Finally, the concluding remarks are given in Section 4. 

## 2. Thermally Induced Driving Moment of the Hollow Torus Motor

In this section, based on the temperature field in a cross section of a rotating hollow torus in steady state, a theoretical model of thermally driven self-sustained rotation is established, and the analytical driving moment is further derived. Meanwhile, the effects of the angular velocity and the radius ratio on the driving moment are discussed. 

### 2.1. Temperature Field in the Steadily Rotating Hollow Torus Motor

**Figure 1:**
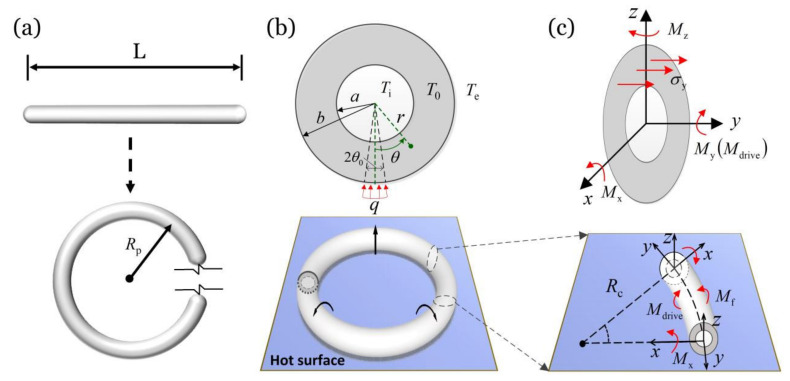
Schematic of a thermally driven rotating hollow torus motor on a hot surface. (**a**) An initially straight hollow rod with length L is curved into a prestressed torus with a curvature radius $R_{p}=L/2\pi$ or curvature κ=2π/L. (**b**) Through a contact area between the hollow torus and the hot surface, a constant heat flux q transfers from the hot surface to the torus. (**c**) Inhomogeneous thermal contraction caused by inhomogeneous temperature field in the hollow torus can generate a driving moment $M_{\mathrm{drive}}$, which causes the hollow torus to invert or evert.

Figure 1 sketches a thermally responsive hollow torus motor which can self-rotate on a hot surface. A hollow straight rod with a length of L is first curved into a prestressed torus with a curvature radius $R_{p}=L/2\pi$ or curvature κ=2π/L, as shown in Figure 1a. Next, the prestressed hollow torus is placed on the hot surface, and a constant heat flux q transfers from the hot surface to the torus through a contact area between the torus and the hot surface, as shown in Figure 1b. a is the inner radius of the torus and b is outer radius of the torus. Because of the inhomogeneous temperature field in the cross section of the torus, the inhomogeneous deformation is induced and then drive the hollow torus to invert or evert. The curvature radius of the torus in steady rotation is $R_{c}$, as shown in Figure 1c. 

The Euler polar coordinate system and Euler rectangular coordinate system established in this paper are shown in Figure 1b,c, respectively. For simplicity, we ignore the Poisson’s effect on the cross section of the hollow torus in the following analysis, and thus the problem in this paper can be reduced to a planar problem. The differential equation that governs the temperature field in a cross section of the hollow torus is given by [27,59,60]

(1) $$\frac{\partial T\left(r,\theta ,t\right)}{\partial t}+\omega \left(t\right)r\frac{\partial T\left(r,\theta ,t\right)}{\partial \theta }=\frac{k}{\rho c}\nabla^{2}T\left(r,\theta ,t\right)$$

where $T\left(r,\theta ,t\right)$ is temperature field in the cross section of the rotating hollow torus, k is the heat conduction coefficient, ρ is the mass density, c is its specific heat, and $\omega \left(t\right)$ is the angular velocity of the hollow torus at a moment t. In the steady rotation, we write $T\left(r,\theta ,t\right)=T\left(r,\theta \right)$ and $\omega \left(t\right)=\omega$.

We assume that except for a small contact area with the hot surface, the rest of the outer surface of the hollow torus is cooled by convection, while the inner surface of the hollow torus is adiabatic. Then, the associated boundary conditions for Equation (1) are [59,60,61]

(2) $$k\frac{\partial T}{\partial r}=\left\{\begin{array}{ll} q; & r=b,\;-\theta_{0}\leq \theta \leq \theta_{0}\\ -h\left(T-T_{e}\right); & r=b,\;\theta_{0}<\theta <2\pi -\theta_{0}\\ 0; & r=a \end{array}\right.$$

where q is the heat flux, $T_{e}$ is the external environment temperature, h is the coefficient of heat transfer, and $\theta_{0}$ is half contact angle.

By defining the following dimensionless parameters $\bar{r}=r/b$, λ=a/b, $\bar{T}=T/T_{e}$, $\bar{q}=qb/kT_{e}$, $\bar{h}=hb/k$, ${\bar{R}}_{n}=R_{n}b$, ${\bar{F}}_{n}=F_{n}b^{2}$, ${\bar{\beta }}_{m}=\beta_{m}b$, and $\bar{\omega }=\omega b^{2}/\psi$ with ψ being the coefficient of thermal diffusivity, the temperature distribution in a cross section of a rotating hollow torus in steady state can be determined by the integral transformation pair as [60,61]

(3) $$\begin{array}{l} \bar{T}\left(\bar{r},\theta \right)=(\theta_{0}\bar{q}+\pi \bar{h})\sum_{m=1}^{\infty }\frac{{\bar{R}}_{0}\left(\lambda ,\bar{h},\bar{r}\right)}{{\bar{F}}_{0}\left(\lambda ,\bar{h}\right)}\\ +2\bar{q}\sum_{n=1}^{\infty }\sum_{m=1}^{\infty }\frac{{\bar{R}}_{n}\left(\lambda ,\bar{h},\bar{r}\right)}{{\bar{F}}_{n}\left(\lambda ,\bar{h}\right)}\frac{\sin n\theta_{0}}{n}\frac{\cos n\theta +{\bar{\eta }}_{n}\left(\lambda ,\bar{h},\bar{\omega }\right)\sin n\theta }{1+\mu_{n}^{2}\left(\lambda ,\bar{h},\bar{\omega }\right)} \end{array}$$

where $\mu_{n}\left(\lambda ,\bar{h},\bar{\omega }\right)=\bar{\omega }n/{\bar{\beta }}_{m}^{2}\left(\lambda ,\bar{h}\right)$, and the functions ${\bar{R}}_{n}\left(\lambda ,\bar{h},\bar{r}\right)$ and ${\bar{F}}_{n}\left(\lambda ,\bar{h}\right)$ are given by

(4) $${\bar{R}}_{n}\left(\lambda ,\bar{h},\bar{r}\right)={\bar{L}}_{n}J_{n}\left({\bar{\beta }}_{m}\bar{r}\right)-{\bar{V}}_{n}Y_{n}\left({\bar{\beta }}_{m}\bar{r}\right)$$

(5) $${\bar{F}}_{n}\left(\lambda ,\bar{h}\right)=B_{e}\left(\lambda ,\bar{h}\right)-\frac{B_{i}\left(\lambda ,\bar{h}\right){\bar{V}}_{n}^{2}}{{\bar{K}}_{n}^{2}}$$

with the expressions of $B_{i}\left(\lambda ,\bar{h}\right)$ and $B_{e}\left(\lambda ,\bar{h}\right)$ being

(6) $${\bar{B}}_{i}\left(\lambda ,\bar{h}\right)={\bar{\beta }}_{m}^{2}\left(\lambda ,\bar{h}\right)-{\left(\frac{n}{\lambda }\right)}^{2}$$

(7) $${\bar{B}}_{e}\left(\lambda ,\bar{h}\right)={\bar{h}}^{2}+{\bar{\beta }}_{m}^{2}\left(\lambda ,\bar{h}\right)-n^{2}$$

and ${\bar{\beta }}_{m}\left(\lambda ,\bar{h}\right)$ is the positive root of the characteristic equation

(8) $${\bar{K}}_{n}{\bar{L}}_{n}-{\bar{V}}_{n}{\bar{W}}_{n}=0$$

where the functions ${\bar{K}}_{n}$, $\;{\bar{L}}_{n}$, $\;{\bar{V}}_{n}$ and ${\bar{W}}_{n}$ are given by

(9) $${\bar{K}}_{n}=\frac{n}{\lambda }J_{n}\left({\bar{\beta }}_{m}\lambda \right)-{\bar{\beta }}_{m}J_{n+1}\left({\bar{\beta }}_{m}\lambda \right)$$

(10) $${\bar{L}}_{n}=\left(n+\bar{h}\right)Y_{n}\left({\bar{\beta }}_{m}\right)-{\bar{\beta }}_{m}Y_{n+1}\left({\bar{\beta }}_{m}\right)$$

(11) $${\bar{V}}_{n}=\left(n+\bar{h}\right)J_{n}\left({\bar{\beta }}_{m}\right)-{\bar{\beta }}_{m}J_{n+1}\left({\bar{\beta }}_{m}\right)$$

(12) $${\bar{W}}_{n}=\frac{n}{\lambda }Y_{n}\left({\bar{\beta }}_{m}\lambda \right)-{\bar{\beta }}_{m}Y_{n+1}\left({\bar{\beta }}_{m}\lambda \right)$$

with $J_{n}$ and $Y_{n}$ being Bessel functions of the first and second kinds, respectively.

**Figure 2:**
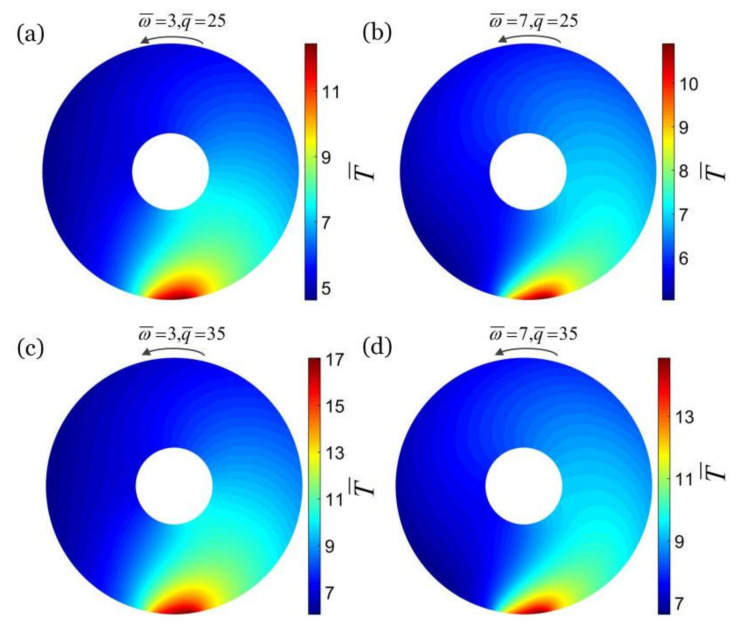
The dimensionless temperature fields in a cross section of a rotating hollow torus in steady state, with different combinations of the dimensionless rotation angular velocity $\bar{\omega }$ and the dimensionless heat flux $\bar{q}$: (**a**) $\bar{\omega }=3,\;\bar{q}=25$, (**b**) $\bar{\omega }=7,\;\bar{q}=25$, (**c**) $\bar{\omega }=3,\;\bar{q}=35$, (**d**) $\bar{\omega }=7,\;\bar{q}=35$. The other parameters are: $\bar{h}=0.2$, λ=0.3 and $\theta_{0}=0.2$. The inhomogeneity of the steady-state temperature field in the cross section of the hollow torus decreases with the increase in rotation angular velocity $\bar{\omega }$ and increases with the increase in heat flux $\bar{q}$.

The typical values of material properties and geometric parameters from accessible experiments [25,27,50] are listed in Table 1, and the estimated values of dimensionless parameters are listed in Table 2. Figure 2 plots the dimensionless temperature fields in a cross section of a rotating hollow torus in steady state, with different combinations of the dimensionless rotation angular velocity $\bar{\omega }$ and the dimensionless heat flux $\bar{q}$: (a) $\bar{\omega }=3,\;\bar{q}=25$, (b) $\bar{\omega }=7,\;\bar{q}=25$, (c) $\bar{\omega }=3,\;\bar{q}=35$, (d) $\bar{\omega }=7,\;\bar{q}=35$, and the other parameters are $\bar{h}=0.3$, λ=0.3 and $\theta_{0}=0.2$. For a given heat flux $\bar{q}$, the temperature difference of the cross section of the hollow torus decreases with the increase in the rotation angular velocity $\bar{\omega }$, and the temperature field tends to be homogeneous. For a given rotation angular velocity $\bar{\omega }$, with the increase in heat flux $\bar{q}$, the temperature difference of the cross section of the hollow torus increases and the inhomogeneity of the temperature field on the cross section increases. These trends are consistent with our expectations.

**Figure 3:**
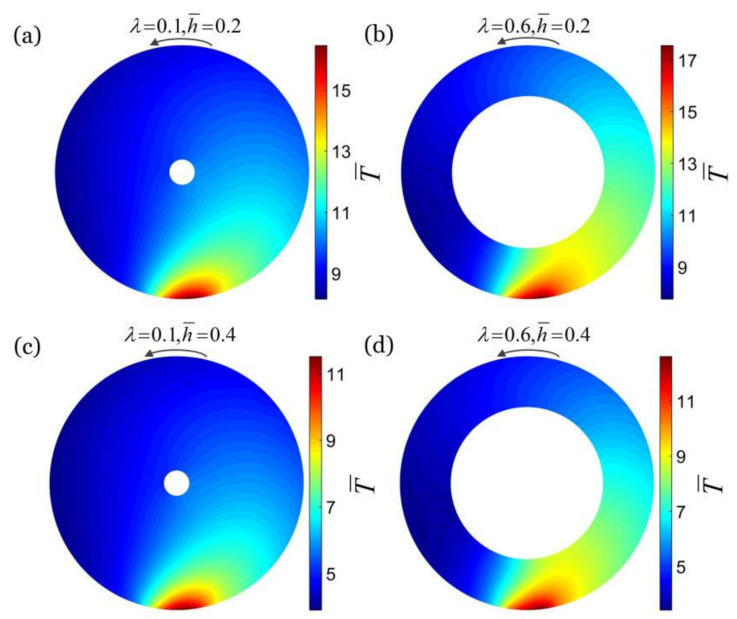
The dimensionless temperature fields in a cross section of a rotating hollow torus in steady state, with different combinations of the radius ratio λ and the dimensionless heat transfer coefficient $\bar{h}$: (**a**) $\lambda =0.1,\;\bar{h}=0.2$, (**b**) $\lambda =0.6,\;\bar{h}=0.2$, (**c**) $\lambda =0.1,\;\bar{h}=0.4$, (**d**) $\lambda =0.6,\;\bar{h}=0.4$. The other parameters are: $\bar{\omega }=4$, $\bar{q}=30$ and $\theta_{0}=0.2$. The inhomogeneity of the steady-state temperature field in the cross section of the hollow torus increases slightly with the increase in the radius ratio λ and decreases with the increase in heat transfer coefficient $\bar{h}$.

Figure 3 plots the dimensionless temperature fields in a cross section of a rotating hollow torus in steady state, with different combinations of the radius ratio and heat transfer coefficient: (a) $\lambda =0.1,\;\bar{h}=0.2$, (b) $\lambda =0.6,\;\bar{h}=0.2$, (c) $\lambda =0.1,\;\bar{h}=0.4$, (d) $\lambda =0.6,\;\bar{h}=0.4$, and the other parameters are: $\bar{\omega }=4$, $\bar{q}=30$ and $\theta_{0}=0.2$. For a given heat transfer coefficient $\bar{h}$, the temperature difference of the cross section of the hollow torus increases slightly with the increase in the radius ratio λ of the hollow torus, and the temperature field tends to be inhomogeneous. This is because that the smaller the radius ratio of the hollow torus λ, the more unfavorable the heat transfer inside the hollow torus, resulting in a lower temperature in a low temperature zone and a higher temperature in a high temperature zone. For a given radius ratio λ, the temperature difference in the cross section of the hollow torus for large heat transfer coefficient decreases with increase in $\bar{h}$, and tends to be homogeneous. It is noted that the temperature difference in the cross section for small heat transfer coefficient increases with the increase in $\bar{h}$ and tends to be inhomogeneous.

### 2.2. Driving Moment for the Rotation of the Hollow Torus Motor

To obtain the thermally induced driving moment for the rotation of the hollow torus, we assume that all the material points in the hollow torus are subjected to uniaxial stress along the hoop direction of the torus, similar to the classic beam theory. For an initially straight hollow rod curved into a prestressed hollow torus with a curvature radius of $R_{p}$ (Figure 1a), and prestrain in the cross section of the hollow torus can be given by

(13) $$\varepsilon_{p}\left(r,\theta \right)=-\frac{r\sin\theta }{R_{p}}$$

We assume that the thermally induced strain $\varepsilon_{T}$ is linearly proportional to the temperature change in the cross section of the hollow torus, i.e.,

(14) $$\varepsilon_{T}\left(r,\theta \right)=C_{T}\left[T\left(r,\theta \right)-T_{e}\right]$$

where, $C_{T}$ is the thermal expansion coefficient of the material. For thermal expansion materials such as PDMS, $C_{T}$ is positive, while for thermal shrinkage materials such as nylon or a monodomain LCE, $C_{T}$ is negative.

With a small deformation assumption, the elastic strain in the cross section can be expressed as

(15) $$\varepsilon_{e}\left(r,\theta \right)=\varepsilon_{p}\left(r,\theta \right)-\varepsilon_{T}\left(r,\theta \right)$$

By using linear thermoelastic model, we can compute the stress $\sigma \left(r,\theta \right)$ on a cross section of the hollow torus along its normal direction as

(16) $$\sigma \left(r,\theta \right)=E\varepsilon_{e}\left(r,\theta \right)$$

where E is the elastic modulus of the material. During steady rotation of the hollow torus, the total bending moment about the x axis in a cross section can be expressed as

(17) $$M_{x}=\int_{r_{1}}^{r_{2}}\int_{0}^{2\pi }\sigma \left(r,\theta \right)r\cos\theta rdrd\theta$$

Since the curvature radius $R_{p}$ is much larger than the outer radius of the hollow torus b, we ignore the influence of temperature on the curvature of the torus in the calculation. Selecting an arbitrary segment from the hollow torus as shown in Figure 1c, we can obtain the total net moment applied to the segment per unit length as

(18) $$M_{\mathrm{drive}}=\kappa M_{x}$$

By defining the following dimensionless parameters ${\bar{C}}_{T}=C_{T}T_{e}$, $\bar{\sigma }=\sigma /E$, ${\bar{M}}_{x}=M_{x}/Eb^{3}$, ${\bar{M}}_{\mathrm{drive}}=M_{\mathrm{drive}}/Eb^{2}$, and $\bar{\kappa }=\kappa b$, and combining Equations (3) and (13)–(17), we can get that the analytical solution of the driving moment of the hollow torus rotating steadily

(19) $${\bar{M}}_{\mathrm{drive}}=-2\pi \bar{\kappa }\bar{q}{\bar{C}}_{T}\sin\theta_{0}\sum_{m=1}^{\infty }\frac{P\left(\lambda ,\bar{h}\right)}{\left[1+\mu_{1}^{2}\left(\lambda ,\bar{h},\bar{\omega }\right)\right]{\bar{F}}_{1}\left(\lambda ,\bar{h}\right)}$$

where the expression of $P\left(\lambda ,\bar{h}\right)$ is

(20) $$\begin{array}{ll} P\left(\lambda ,\bar{h}\right) & =\lambda^{2}J_{2}\left({\bar{\beta }}_{m}\lambda \right)Y_{2}\left({\bar{\beta }}_{m}\lambda \right)\\ & +J_{2}\left({\bar{\beta }}_{m}\right)\left\{\frac{{\bar{\beta }}_{m}}{2}\left[\mathrm{MeijerG}\left(\left\{\left\{-\frac{1}{2}\right\},\left\{-1\right\}\right\},\left\{\left\{-\frac{1}{2},\frac{1}{2}\right\},\left\{-\frac{3}{2},-1\right\}\right\},\frac{{\bar{\beta }}_{m}^{2}}{4}\right)\right.\right.\\ & \left.\left.-\lambda^{3}\mathrm{MeijerG}\left(\left\{\left\{-\frac{1}{2}\right\},\left\{-1\right\}\right\},\left\{\left\{-\frac{1}{2},\frac{1}{2}\right\},\left\{-\frac{3}{2},-1\right\}\right\},\frac{{\bar{\beta }}_{m}^{2}}{4}\right)\right]-Y_{2}\left({\bar{\beta }}_{m}\right)\right\} \end{array}$$

where $\mathrm{MeijerG}\left[\left\{\left\{s_{1}\ldots s_{n}\right\},\left\{s_{n+1}\ldots s_{p}\right\}\right\},\left\{\left\{t_{1}\ldots t_{m}\right\},\left\{s_{m+1}\ldots s_{q}\right\}\right\},z\right]$ is the Meijer G function.

**Figure 4:**
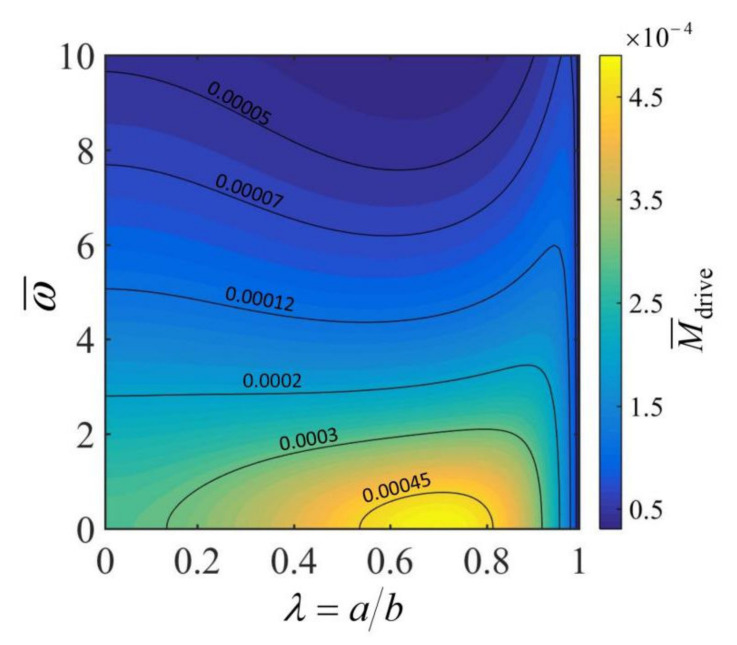
The effects of the dimensionless rotation angular velocity $\bar{\omega }$ and the radius ratio of the hollow torus λ on the dimensionless driving moment ${\bar{M}}_{\mathrm{drive}}$. The parameters are $\bar{h}=0.3$, $\bar{\kappa }=0.025$, ${\bar{C}}_{T}=0.005$, $\bar{q}=30$ and $\theta_{0}=0.2$. For a given angular velocity $\bar{\omega }$, there exists an optimal radius ratio λ that maximizes the driving moment ${\bar{M}}_{\mathrm{drive}}$.

Figure 4 plots the effects of the dimensionless rotation angular velocity $\bar{\omega }$ and the radius ratio of the hollow torus λ on the dimensionless driving moment ${\bar{M}}_{\mathrm{drive}}$ for $\bar{h}=0.3$, $\bar{\kappa }=0.025$, ${\bar{C}}_{T}=0.005$, $\bar{q}=30$ and $\theta_{0}=0.2$. It can be seen that for a given radius ratio λ, the driving moment ${\bar{M}}_{\mathrm{drive}}$ decreases with the increase in the rotation angular velocity $\bar{\omega }$. This can be understood from Figure 2. The inhomogeneity of the steady-state temperature field in the cross section of the hollow torus decreases with the increase in rotation angular velocity $\bar{\omega }$, which causes the reduction of the driving moment ${\bar{M}}_{\mathrm{drive}}$. For a given rotation angular velocity $\bar{\omega }$, the driving moment ${\bar{M}}_{\mathrm{drive}}$ of the hollow torus increases and then decreases with increase in the radius ratio λ. This is because that the inhomogeneity of the temperature field in the cross section of the hollow torus increases slightly with the increase in λ (as shown in Figure 3), and for a smaller λ, the increased driving moment due to the increase in temperature field inhomogeneity is sufficient to compensate for the loss of driving moment due to the decrease of cross-sectional area. For a given rotation angular velocity $\bar{\omega }$, there exists an optimal radius ratio of the hollow torus λ that maximizes the driving moment ${\bar{M}}_{\mathrm{drive}}$. However, the actual maximum rotation angular velocity depends on the competition between the driving moment and the friction moment.

## 3. Self-Sustained Rotation of the Hollow Torus Motor on a Hot Surface

Based on the driving moment in Equation (19), the equilibrium equation during steady rotation of the hollow torus is further derived in this section. Then, the effects of the radius ratio, thermal expansion coefficient, heat flux, contact angle, curvature of the torus, heat transfer coefficient and sliding friction coefficient on the rotation angular velocity of the hollow torus are studied in detail, and the critical value for triggering the self-sustained rotation is found. Furthermore, the dependence of the energy efficiency on the radius ratio is also investigated.

### 3.1. Equilibrium Equations

During steady rotation, the surface of the hollow torus is also subjected to a sliding frictional force between the hollow torus and the hot surface. Selecting an arbitrary segment of length from the hollow torus, we can obtain the magnitude of the frictional force applied to the segment per unit length as

(21) $$F_{f}=C_{f}\rho g\pi \left(b^{2}-a^{2}\right)$$

where $C_{f}$ is the sliding friction coefficient between the hollow torus and the hot surface, and g is the gravitational acceleration. The friction moment on the hollow torus per unit length can be given as

(22) $$M_{f}=F_{f}b$$

During steady rotation, the driving moment is equal to the friction moment. Therefore, the equilibrium equation for the steady rotation can be derived from Equations (19) and (22) as

(23) $$\frac{C_{f}\rho gk}{E\kappa qbC_{T}\sin\theta_{0}}=F\left(\lambda ,\bar{h},\bar{\omega }\right)$$

where

(24) $$F\left(\lambda ,\bar{h},\bar{\omega }\right)=\frac{2}{1-\lambda^{2}}\sum_{m=1}^{\infty }\frac{P\left(\lambda ,\bar{h}\right)}{\left[1+\mu_{1}^{2}\left(\lambda ,\bar{h},\bar{\omega }\right)\right]{\bar{F}}_{1}\left(\lambda ,\bar{h}\right)}$$

From Equation (23), we can see that although there are many parameters related to the angular velocity $\bar{\omega }$, including $C_{f}$, k, ρ, E, b, κ, $C_{T}$, $\theta_{0}$, a and h, we only need to analyze the effect of three parameters: radius ratio λ, heat-transfer coefficient $\bar{h}$ and dimensionless parameter $\Pi =C_{f}\rho gk/E\kappa qbC_{T}\sin\theta_{0}$ on the rotation angular velocity.

### 3.2. Angular Velocity of the Self-Rotation of the Hollow Torus Motor

**Figure 5:**
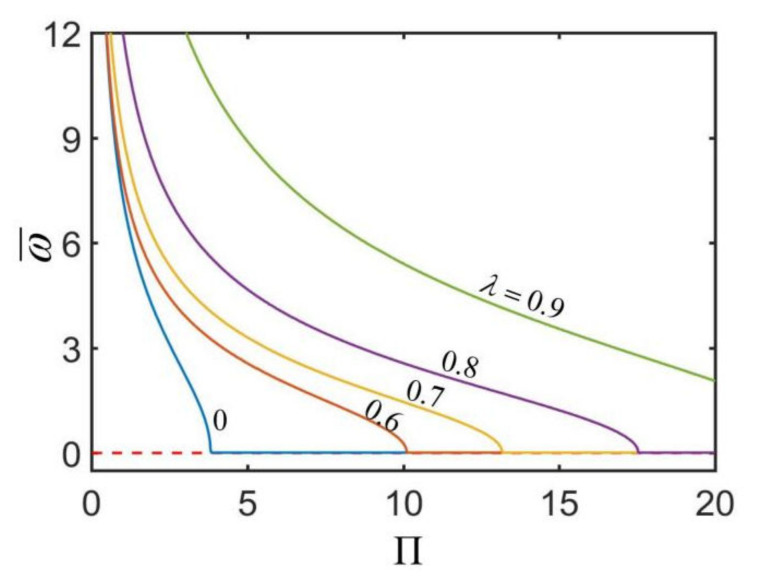
The effect of the dimensionless parameter Π on the dimensionless rotation angular velocity $\bar{\omega }$ of the hollow torus for $\bar{h}=0.3$. The rotation angular velocity $\bar{\omega }$ decreases with the increase in Π. There is a critical $\Pi_{\mathrm{crit}}$ for triggering the rotation of the hollow torus, and $\Pi_{\mathrm{crit}}$ increases with the increase in the radius ratio.

Figure 5 plots the effect of the dimensionless parameter Π on the dimensionless rotation angular velocity $\bar{\omega }$ of the hollow torus for different radius ratios. In the computation, we set $\bar{h}=0.3$. The calculations show that there is a critical $\Pi_{\mathrm{crit}}$ for triggering the rotation of the hollow torus, and $\Pi_{\mathrm{crit}}$ increases with the increase in the radius ratio. It can be seen that with the increase in Π, the rotation angular velocity $\bar{\omega }$ first decreases to zero and then keeps at zero. This is because that the increasing Π decreases the driving moment ${\bar{M}}_{\mathrm{drive}}$ while increases the frictional moment ${\bar{M}}_{f}$, as shown in Equation (23). Considering that $\Pi =C_{f}\rho gk/E\kappa qbC_{T}\sin\theta_{0}$, we can also conclude that the rotation angular velocity $\bar{\omega }$ of hollow torus increases with the increase in the heat flux q, contact angle $\theta_{0}$, thermal expansion coefficient $C_{T}$, the outer radius b, and curvature of hollow torus κ, the elastic modulus of the material E, and decreases with the increase in the sliding friction coefficient $C_{f}$, mass density ρ and heat conduction coefficient k. The effects of these parameters on the rotation angular velocity of the hollow torus are consistent with that of the solid torus on a hot surface [50].

**Figure 6:**
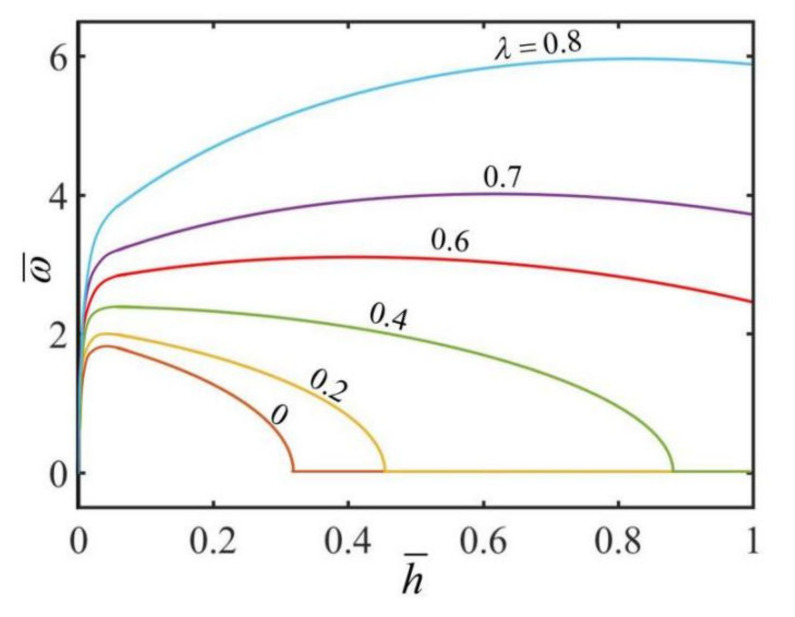
The effect of the dimensionless heat transfer coefficient $\bar{h}$ on the dimensionless rotation angular velocity $\bar{\omega }$ of the hollow torus for Π=4. The rotation angular velocity $\bar{\omega }$ increases and then decreases with the decrease of the heat transfer coefficient.

Figure 6 plots the effect of the dimensionless heat transfer coefficient $\bar{h}$ on the dimensionless rotation angular velocity $\bar{\omega }$ of the hollow torus for different radius ratio. In the computation, we set Π=4. The result shows that for a given radius ratio λ, there exists a critical thermal expansion coefficient ${\bar{h}}_{\mathrm{crit}}$ for triggering the self-rotation, and the critical value increases with the increase in the radius ratio. For a given radius ratio λ, the rotation angular velocity first increases and then decreases with the increase in the coefficient of heat transfer. It can be understood that for small heat transfer coefficients, the inhomogeneity of the steady-state temperature field in the cross section of the hollow torus increases with the increase in heat transfer coefficient $\bar{h}$, while for large heat transfer coefficient, the inhomogeneity of the steady-state temperature field decreases with the increase in heat transfer coefficient $\bar{h}$.

**Figure 7:**
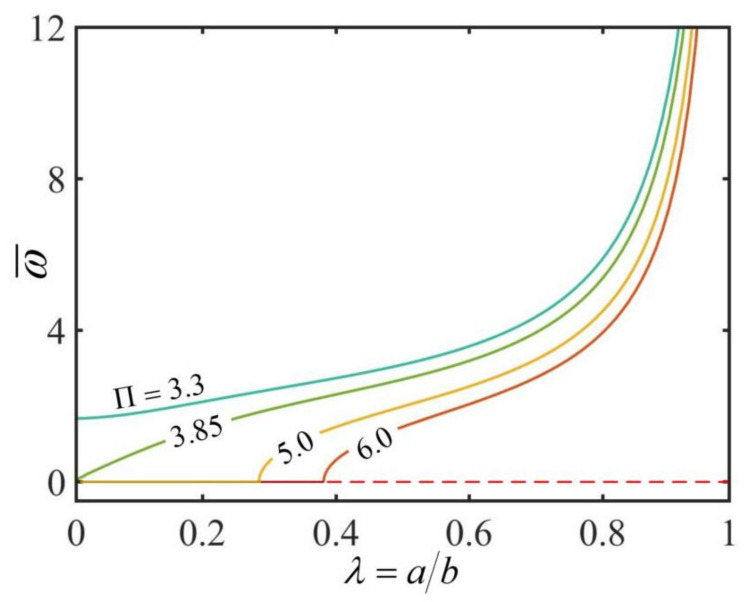
The effect of the radius ratio λ on the dimensionless rotation angular velocity $\bar{\omega }$ of the hollow torus for $\bar{h}=0.3$. The rotation angular velocity $\bar{\omega }$ increases with the increase in the radius ratio λ.

Figure 7 plots the effect of the radius ratio λ on the dimensionless rotation angular velocity $\bar{\omega }$ of the hollow torus for different Π. In the computation, we set $\bar{h}=0.3$. The results show that for a given Π<3.85, the rotation angular velocity $\bar{\omega }$ increases with the increase in the radius ratio λ from zero. For Π>3.85, there exists a critical radius ratio $\lambda_{\mathrm{crit}}$ for the self-rotation of the torus, which increases with the increase in the Π. With the increase in the radius ratio from $\lambda_{\mathrm{crit}}$, the rotation angular velocity increases. This is because that as the radius ratio increases, the friction moment ${\bar{M}}_{f}$ decreases by more percentage than the driving moment ${\bar{M}}_{\mathrm{drive}}$. This result implies we can improve the thermo-mechanical energy conversion efficiency of the hollow torus by increasing the radius ratio λ.

### 3.3. Energy Efficiency of the Self-Rotating Hollow Torus Motor

The self-rotating hollow torus motor studied in this paper has the potential to be explored as a motor or an energy harvester. In the practical applications, the energy conversion efficiency highly depend on the specific energy conversion processes. In the current study, during the steady self-oscillation of the hollow torus, the thermal energy harvested by the motor compensates the damped energy. Therefore, we can regard the damped energy as the effective work that the system does to the external devices, and the effective power done by the motor of unit length is $P_{e}=M_{f}\omega$. Meanwhile, the thermal power from the hot surface to the torus of unit length during the steady rotation can be expressed as $P_{t}=2q\theta_{0}b$. The energy efficiency of the self-rotating hollow torus is defined to be the ratio of the effective power to the thermal power, and can be given by combining Equations (22) and (23) as

(25) $$\eta =\frac{C_{f}\rho g\pi \left(b^{2}-a^{2}\right)\omega }{2q\theta_{0}}$$

**Figure 8:**
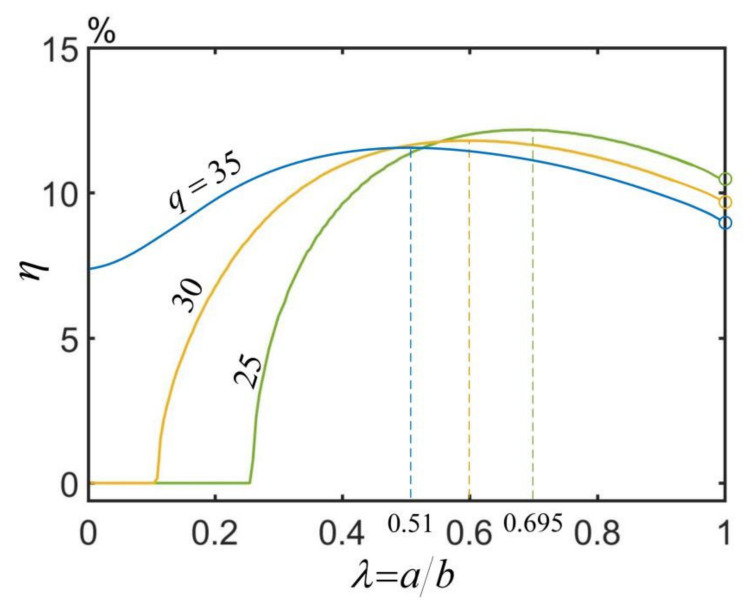
Dependences of the energy efficiency η on the radius ratio λ for three different dimensionless heat fluxes $\bar{q}$. The parameters are $\bar{h}=0.3$, $\bar{\kappa }=0.02$, ${\bar{C}}_{T}=0.005$, $C_{f}=1.2$ and $\theta_{0}=0.2$. There exists an optimal radius ratio that maximizes the energy efficiency of the torus, and the optimal radius ratio decreases as the heat flux increases.

We can see from Equation (25) that there are many parameters affecting the energy efficiency η, including $T_{e}$, $C_{f}$, k, ρ, q, b, κ, $C_{T}$, $\theta_{0}$, a and h. Figure 8 plots the dependences of the energy efficiency η on the radius ratio λ for three different dimensionless heat fluxes $\bar{q}$. In the computation, we choose the typical values of the parameters as shown in Table 1, and the dimensionless parameters are calculated to be $\bar{h}=0.3$, $\bar{\kappa }=0.02$, ${\bar{C}}_{T}=0.005$, $C_{f}=1.2$ and $\theta_{0}=0.2$. For large $\bar{q}=35$, the energy efficiency first increases and then decreases with the increase in the radius ratio. For small $\bar{q}$, there exists a critical radius ratio $\lambda_{\mathrm{crit}}$ for triggering the self-rotation, which increases with the decrease of $\bar{q}$. Similarly, the energy efficiency first increases and then decreases when the radius ratio increases from $\lambda_{\mathrm{crit}}$. Therefore, there exists an optimal radius ratio that maximizes the energy efficiency of the hollow torus motor, and the optimal radius ratio decreases as the heat flux increases as shown in Figure 8. From Figure 8, the maximum energy efficiency, optimal radius ratio, energy efficiency of solid torus and energy efficiency improvement, with three different heat fluxes $\bar{q}$ are listed in Table 3. It is shown that the energy efficiency for $\bar{q}=25$ increases from 0% to 12.17%. This result implies that the energy efficiency can be augmented by adjusting the radius ratio of the hollow torus in practical applications.

## 4. Conclusions

Self-oscillating systems based on thermally responsive polymer materials have the advantages of simple structure, strong practicability and sustainability, and can realize heat-mechanical transduction in a steady ambient temperature field, which have huge application potential in the field of micro-active machines, micro-robotics and energy harvesters. In this article, by establishing a theoretical model of heat-driven self-sustained rotation of a hollow torus motor, we obtain the analytical solution of the heat-induced driving moment, and then provide the equilibrium equation for the steady rotation of the motor. Through detailed calculations, it was found that the rotation angular velocity of the motor increases with the increase in the radius ratio, heat flux, contact angle, thermal expansion coefficient, curvature, and the elastic modulus, and decreases with the increase in the sliding friction coefficient, mass density, heat transfer coefficient and heat conduction coefficient. In addition, for a given heat flux there exists an optimal radius ratio that maximizes the energy efficiency of the hollow torus motor. In the future, it is worthwhile to carry out the corresponding experiments to verify the theoretical predictions. Meanwhile, the effects of viscoelasticity and the cross-sectional shape on the rotation of the hollow torus motor also need to be further investigated. The self-rotating hollow torus motor studied in this paper has the potential for energy harvesting, mass transport and lifting heavy objects.

## Figures and Tables

**Table 1:** Material properties and geometric parameters.

Parameter	Definition	Value	Units
*T*_e_	temperature field	10	${}^{\circ }C$
a	internal radius	0~1	mm
b	external radius	1	mm
h	heat transfer coefficient	10~20	$W/m^{2}{}^{\circ }C$
*Ψ*	coefficient of thermal diffusivity	$1.2\times 10^{-6}$	$m^{2}/s$
*k*	heat conduction coefficient	0.05~0.1	$W/m^{\circ }C$
*q*	heat flux	$0\sim 20\times 10^{3}$	$W/m^{2}$
*ω*	rotation angular velocity	0~4π	$s^{-1}$
κ	curvature of the prestressed torus	0~20	$m^{-1}$
$C_{T}$	thermal expansion coefficient	$5\times 10^{-4}$	$1/{}^{\circ }C$
E	elastic modulus of the material	5	MPa
ρ	mass density	$1.3\times 10^{3}$	$\mathrm{kg}/m^{3}$
g	gravitational acceleration	10	$m/s^{2}$
$C_{f}$	sliding friction coefficient	0.6~1.2	

**Table 2:** Dimensionless parameters.

Parameter	$\bar{q}$	$\bar{h}$	*λ*	$\bar{\omega }$	${\bar{C}}_{T}$	$\bar{\kappa }$	Π
Value	0~40	0.1~0.6	0~1	0~12	0.005	0~0.05	0~50

**Table 3:** Energy efficiency improvement of the hollow torus motor.

Dimensionless Heat Flux $\bar{q}$	Optimal Radius Ratio λ	Energy Efficiency of Solid Torus	Maximum Energy Efficiency	Energy Efficiency Improvement
35	0.510	7.41%	11.56%	4.15%
30	0.598	0%	11.82%	11.82%
25	0.695	0%	12.17%	12.17%
